# Concurrent smoking and alcohol consumers had higher triglyceride glucose indices than either only smokers or alcohol consumers: a cross-sectional study in Korea

**DOI:** 10.1186/s12944-021-01472-2

**Published:** 2021-05-11

**Authors:** Wonhee Baek, Ji-Won Lee, Hye Sun Lee, Donghee Han, Su-Yeon Choi, Eun Ju Chun, Hae-Won Han, Sung Hak Park, Jidong Sung, Hae Ok Jung, Hyangkyu Lee, Hyuk-Jae Chang

**Affiliations:** 1 Department of Nursing, Yonsei University Graduate School, Seoul, Republic of Korea; 2 Department of Nursing, Kyungnam University College of Health Sciences, Changwon, Republic of Korea; 3 Department of Family Medicine, Yonsei University College of Medicine Gangnam Severance Hospital, Seoul, Republic of Korea; 4 Biostatistics Collaboration Unit, Department of Research Affairs, Yonsei University College of Medicine, Seoul, Republic of Korea; 5 Department of Imaging and Medicine, Cedars Sinai Medical Center, Los Angeles, CA USA; 6 Division of Cardiology, Seoul National University Healthcare System Gangnam Center, Seoul National University College of Medicine, Seoul, Republic of Korea; 7 Department of Radiology, Seoul National University Bundang Hospital, Seoul, Republic of Korea; 8 Department of Internal Medicine, Gangnam Heartscan Clinic, Seoul, Republic of Korea; 9 Department of Radiology, Gangnam Heartscan Clinic, Seoul, Republic of Korea; 10 Division of Cardiology, Department of Medicine, Sungkyunkwan University School of Medicine, Heart Stroke and Vascular Institute, Samsung Medical Center, Seoul, Republic of Korea; 11 Division of Cardiology, Cardiovascular Center, College of Medicine, Seoul St. Mary’s Hospital, The Catholic University of Korea, Seoul, Republic of Korea; 12 Mo-Im Kim Nursing Research Institute, College of Nursing, Yonsei University, 50-1, Yonsei-ro, Seodaemun-gu, Seoul, 03722 Republic of Korea; 13 Division of Cardiology, Severance Cardiovascular Hospital, Yonsei University College of Medicine, Yonsei University Health System, Seoul, Republic of Korea

**Keywords:** Alcohol consumption, Cross-sectional study, Korean, Life-style modification, Smoking, Insulin resistance, Triglyceride glucose index

## Abstract

**Background:**

The triglyceride glucose (TyG) index is a noninsulin-based marker for insulin resistance (IR) in general practice. Although smoking and heavy drinking have been regarded as major risk factors for various chronic diseases, there is limited evidence regarding the combined effects of smoking and alcohol consumption on IR. This study aimed to investigate the relationship between the TyG index and smoking and alcohol consumption using two Korean population-based datasets.

**Methods:**

This study included 10,568 adults in the Korean National Health and Nutrition Examination Survey (KNHANES) and 9586 adults in the Korean Initiatives on Coronary Artery Calcification (KOICA) registry datasets. Multivariate logistic analysis was conducted to explore the relationship between smoking and alcohol consumption and the TyG index. To assess the predictive value of smoking and alcohol consumption on high TyG index, the area under the curve (AUC) were compared and net reclassification improvement (NRI) and integrated discrimination improvement (IDI) analyses were derived.

**Results:**

The combined effect of smoking and alcohol consumption was an independent risk factor of a higher TyG index in the KNHANES (adjusted odds ratio: 4.33, *P* < .001) and KOICA (adjusted odds ratio: 1.94, *P* < .001) datasets. Adding smoking and alcohol consumption to the multivariate logistic models improved the model performance for the TyG index in the KNHANES (AUC: from 0.817 to 0.829, *P* < .001; NRI: 0.040, *P* < .001; IDI: 0.017, *P* < .001) and KOICA (AUC: from 0.822 to 0.826, *P* < .001; NRI: 0.025, *P* = .006; IDI: 0.005, *P* < .001) datasets.

**Conclusions:**

Smoking and alcohol consumption were independently associated with the TyG index. Concurrent smokers and alcohol consumers were more likely to have a TyG index that was ≥8.8 and higher than the TyG indices of non-users and those who exclusively consumed alcohol or smoking tobacco.

## Background

Insulin resistance (IR) is defined as a metabolic state in which the responsiveness of the target tissues to insulin concentrations is reduced [1], and IR plays a crucial role in various metabolic diseases [2–4]. Therefore, early detection of IR could be instrumental in identifying chronic diseases and establish effective disease management strategies. The hyperinsulinemic-euglycemic clamp (HIEC) is considered the gold standard method for determining IR [5]; however, it is a time-consuming and invasive method with limited applicability to the general population. For this reason, several IR surrogate indices, such as homeostatic model assessment for insulin resistance (HOMA-IR), quantitative insulin sensitivity check index (QUICKI), the McAuley index [6], triglyceride (TG): high density lipoprotein (HDL) cholesterol ratio, lipid accumulation product (LAP), and visceral adiposity index (VAI) [7], have been used previously. Recently, the triglyceride glucose (TyG) index has been suggested to be useful for estimating IR risk [4, 8–10]; furthermore, it is regarded as a “pan-cardiovascular disease (CVD) risk marker” [4, 10, 11]. The TyG index is a noninsulin-based index and is a simple, fast, and inexpensive surrogate, making it advantageous for use in general practice [8].

Cigarette smoking and heavy alcohol consumption have been demonstrated to be major causes of mortality and morbidity in the past few decades. Additionally, this combination can lead to synergistic adverse effects, particularly in the incidence of metabolic syndrome, neurocognitive disorders, and cancers [12–14], and can aggravate the risk of death from CVDs [15]. Smoking and alcohol consumption habits tend to occur concomitantly [16] and the concerns warranted by their synergistic effect on health cannot be overemphasized. The recently growing evidence base suggests that heavy alcohol consumption and smoking impairs insulin action and causes IR [17–20]. However, inconsistent results have been previously reported [21, 22], and limited evidence is available regarding the relationship between IR and concurrent smoking and alcohol consumption in the general population. Furthermore, no studies have been reported to examine the combined effects of smoking and alcohol on IR using the TyG index as an IR marker.

The primary objective of this study was to determine how the concurrent smoking and alcohol habits affect TyG index in a healthy population. The hypothesis was that co-users of cigarettes and alcohol have a high TyG index (≥8.8) that is greater than the TyG index of non-users and those who exclusively consumed alcohol or smoked. The secondary objective of this study was to find out whether the discrimination ability of TyG index is significantly improved when smoking and alcohol habits are added to the traditional risk factors.

## Methods

### Aim

The aim of this study was to determine how the concurrence of smoking and alcohol consumption affected the TyG index of a healthy population of Korean adults.

### Study design

This research followed the Strengthening the Reporting of Observational Studies in Epidemiology (STROBE) guideline [23]. The study had a cross-sectional design and used two Korean population-based datasets.

### Study population

This study consisted of two different population-based cohorts: the 2013–2018 Korean National Health and Nutrition Examination Survey (KNHANES) and the Korean Initiatives on Coronary Artery Calcification (KOICA) registry. The KNHANES is a nationwide cross-sectional survey conducted annually by the Korea Centers for Disease Control and Prevention (KCDC), and participants are hierarchically extracted to represent the population of Korea. Sampling was conducted using a multi-stage clustered probability design based on sex, age, and geographical area [24]. The KOICA registry is a retrospective, multicenter, and observational registry. It consists of self-reported data of asymptomatic patients who received general health examinations at six public healthcare centers in Korea from December 2012 to August 2016 [25]. To be included in the study, subjects had to be 19 years of age or older and have no history of diabetes mellitus, cardiovascular disease, and cancer. Subjects with incomplete data were excluded.

### Bias

KNHANES was a nationwide representative cross-sectional survey, whereas the KOICA registry comprised retrospective data obtained from people who had undergone health examination. The sampling strategy of the latter could contribute to a selection bias. Moreover, in the absence of significant covariates, such as cholesterol levels, blood pressure, and glucose, in the data, biases might exist because of their exclusion from the analysis.

### Assessment of the TyG index

The TyG index was determined using the following formula: ln (triglycerides [mg/dL] × glucose [mg/dL]/2).

Based on a study that predicted type 2 diabetes mellitus (T2DM) onset after 4 years and used a TyG index of 8.8 as the threshold value [26], those with a TyG index of 8.8 or higher were allocated to the high TyG group, while those with a TyG index less than 8.8 were allocated to the normal TyG group. In this study, the third quantile of the TyG index was 8.54 and 8.58 in the KNHANES and KOICA registry, respectively.

### Assessment of smoking and alcohol

Data regarding the participants’ smoking and alcohol consumption habits were collected via self-reported questionnaires. In the KNHANES, depending on the answers to three questions; “Have you ever drank more than one drink in your life?”, “How often do you drink alcohol?”, and “How much alcohol do you drink at one time?”, a participant was considered a heavy drinker if their average alcohol consumption was more than 30 g per day [27]; otherwise, a participant was considered a normal drinker (includes “never drinkers”). In the KOICA registry, depending on the answer to the question “How many times a week do you drink alcohol?”, a participant was considered a heavy drinker if they drank more than three or four times a week; otherwise, they were considered a normal drinker. Current smoking status was classified as “non-smoker” (never smoked cigarettes or smoked < 100 cigarettes during their lifetimes but were currently non-smokers) and “current smoker” (smoked ≥100 cigarettes in their lifetimes and were currently smokers). Depending on their smoking and alcohol consumption, the participants were divided into four groups: those who were both non-smokers and normal drinkers were “non-users,” those who were non-smokers but heavy drinkers were “drinkers,” those who were current smokers but normal drinkers were “smokers,” and those who were both current smokers and heavy drinkers were “co-users.”

### Covariates

Household income and final education were only investigated in the KNHANES, with the former presented as quartiles and the latter divided into the categories of elementary school, middle school, high school, and university. Depending on the answer to the question “How much exercise do you usually do per day?”, regular exercise was defined as exercise for more than 20 min at a time and more than three times a week in the KNHANES [28]. In the KOICA registry, depending on the answer to the question “How many times a week do you exercise?”, regular exercise was defined as regular if the participants exercised more than three or four times a week.

Blood pressure (BP) was defined as the average of the last two of the three values measured manually in the KNHANES and was assessed using an automatic manometer after a minimum 5-min rest in the KOICA registry. Body mass index (BMI) was calculated as the body weight divided by height squared (kg/m^2^). Waist circumference (WC) was measured at the umbilicus with the patient in the standing position.

Blood samples were obtained from an antecubital vein after a minimum 8-h fasting period in accordance to the relevant guidelines and regulations. Collected samples were tested for fasting glucose, total cholesterol (TC), triglycerides (TGs), HDL cholesterol, and low-density lipoprotein (LDL) cholesterol.

Hypertension was defined as a systolic blood pressure (SBP) ≥140 mmHg, diastolic blood pressure (DBP) ≥90 mmHg, or a previous diagnosis of hypertension [29]. Diabetes was defined as fasting glucose ≥126 mg/dL or HbA1c ≥6.5% [30], or a previous diagnosis of diabetes. Dyslipidemia was defined as a previous diagnosis of dyslipidemia, or one of the following four criteria: (1) hypercholesterolemia (serum TC ≥240 mg/dL), (2) hypertriglyceridemia (serum TG ≥200 mg/dL), (3) hyper-LDL cholesterolemia (serum LDL cholesterol ≥160 mg/dL) [31], or (4) hypo-HDL cholesterolemia (serum HDL cholesterol < 40 mg/dL in males, 50 mg/ dL in females) [32].

### Statistical analyses

The statistical analyses were performed using R, version 4·0·0 [33]. To compare the demographic and metabolic characteristics, an independent *t* test, rank sum tests, and a chi-square test were used according to characteristics of the variables. Density plots were used to confirm the distribution of TyG indices according to the four user groups. The differences in the TyG indices according to smoking and alcohol consumption were determined using an analysis of variance, with the demographic and metabolic characteristics values as covariates (ANCOVA). Multivariate analysis was performed to investigate whether smoking and alcohol consumption predicted a high TyG index. ANCOVA and multivariate logistic regression were adjusted for age, sex, final education, SBP, WC, TC, HDL, and regular exercise in the KNHANES, and were adjusted for age, sex, SBP, BMI, TC, HDL, and regular exercise in the KOICA registry. A receiver operating characteristic (ROC) curve analysis was performed to evaluate the predictability of the high TyG index of smoking and alcohol consumption. The overall predictive accuracy was quantified using the area under the ROC curve (AUC), and differences in the AUCs were compared using the Delong method [34]. In addition, net reclassification improvement (NRI) and integrated discrimination improvement (IDI) analyses were performed to evaluate the predictability of smoking and alcohol consumption [35]. Statistical significance was defined as a *P*-value <.05.

## Results

**Fig. 1:**
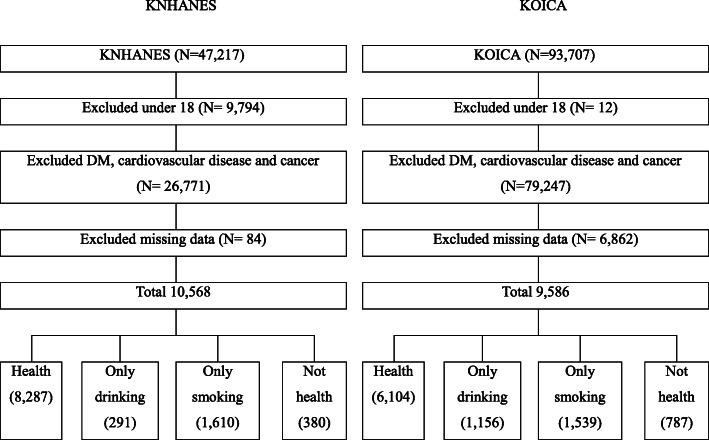
Participant selection flow chart. KNHANES, Korean National Health and Nutrition Examination Survey; KOICA, Korean Initiatives on Coronary Artery Calcification; DM; diabetes mellitus

The participant selection flow chart is shown in Fig. 1. From a total of 47,217 participants in the baseline KNHANES, this study selected 10,568 participants by excluding patients who met the following criteria: (1) 18 years and younger (*n* = 9794), (2) diagnosed with diabetes, CVD, or cancer (*n* = 26,771), and (3) had missing data (*n* = 84). From a total of 93,707 participants in the KOICA registry baseline survey, we selected 9586 participants by excluding patients who met the following criteria: (1) 18 years and younger (*n* = 12), (2) diagnosed with diabetes, a CVD, or cancer (*n* = 79,247), and (3) had missing data (*n* = 6862).

### Participants’ clinical characteristics

In the KNHANES, the mean age of the 10,568 participants was 40.6 ± 14.3 years (19–80 years). A total of 4394 men (41.6%) and 6174 women (58.4%) were included in the analysis. University was the highest education level in 48.6% of the participants, 35.6% were in the fourth income quartile, and 74.8% did not exercise regularly. When the participants were divided into the four smoking and alcohol consumption groups, 78.4% were non-users, 2.8% were drinkers, 15.2% were smokers, and 3.6% were co-users.

In the KOICA registry, the mean age of the 9586 participants was 47.6 ± 9.3 years (19–86 years). A total of 5972 men (62.3%) and 3614 women (37.7%) were included in the analysis. Most participants (59.0%) did not exercise regularly. When the participants were divided into the four smoking and alcohol consumption groups, 63.7% were non-users, 12.1% were drinkers, 16.1% were smokers, and 8.2% were co-users.

The clinical characteristics of the high and normal TyG index groups are shown in Table 1. There were statistically significant differences between the two groups in terms of age, sex, final education, and smoking and alcohol consumption in the KNHANES. In the KOICA registry, statistically significant differences existed between the two groups in terms of sex, regular exercise, and smoking and alcohol consumption.

**Table 1:** Clinical characteristics of the high and normal TyG index groups

	KNHANES (2013–2018)		*P*	KOICA registry (2012–2016)		*P*
	Normal TyG (*N* = 9414)	High TyG (*N* = 1154)		Normal TyG (*N* = 8417)	High TyG (*N* = 1169)	
Age	40.1 ± 14.2	44.8 ± 14.0	<.001	47.6 ± 9.4	48.0 ± 9.1	.102
Sex			<.001			<.001
Female	5846 (62.1)	328 (28.4)		3494 (41.5)	120 (10.3)	
Male	3568 (37.9)	826 (71.6)		4923 (58.5)	1049 (89.7)	
Final education			<.001			
Elementary school	599 (6.4)	107 (9.3)				
Middle school	515 (5.5)	71 (6.2)				
High school	3678 (39.1)	463 (40.1)				
University	4622 (49.1)	513 (44.5)				
Household Income			.106			
Quartile 1	858 (9.1)	127 (11)				
Quartile 2	2156 (22.9)	270 (23.4)				
Quartile 3	3025 (32.1)	374 (32.4)				
Quartile 4	3375 (35.9)	383 (33.2)				
Regular exercise			.259			.010
No	7022 (74.6)	879 (76.2)		4926 (58.5)	731 (62.5)	
Yes	2392 (25.4)	275 (23.8)		3491 (41.5)	438 (37.5)	
Smoking and alcohol			<.001			<.001
Non-user	7595 (80.7)	692 (60)		5517 (65.5)	587 (50.2)	
Drinker	227 (2.4)	64 (5.5)		979 (11.6)	177 (15.1)	
Smoker	1324 (14.1)	286 (24.8)		1292 (15.4)	247 (21.2)	
Co-user	268 (2.8)	112 (9.7)		629 (7.5)	158 (13.5)	

The high TyG index group comprises subjects with a TyG index ≥8.8

Values are presented as the mean and standard deviation or number (%)

*KNHANES* Korean National Health and Nutrition Examination Survey, *KOICA* Korean Initiatives on Coronary Artery Calcification, *TyG* Triglyceride glucose

### Participants’ metabolic characteristics

The metabolic characteristics of the participants in the high and normal TyG index groups are shown in Table 2. SBP, DBP, BMI, glucose, TC, TG, and LDL levels were significantly higher and the HDL levels were significantly lower in the high TyG group in both the KNHANES and KOICA registry (*P* < .001).

**Table 2:** Metabolic characteristics of the high and normal TyG index groups

	KNHANES (2013–2018)		*P*	KOICA registry (2012–2016)		*P*
	Normal TyG (*N* = 9414)	High TyG (*N* = 1154)		Normal TyG (*N* = 8417)	High TyG (*N* = 1169)	
SBP	109.1 ± 10.9	115.1 ± 10.2	<.001	113.3 ± 12.1	116.9 ± 11.2	<.001
DBP	71.8 ± 7.9	76.2 ± 7.4	<.001	69.3 ± 9.4	72.7 ± 8.7	<.001
BMI	22.2 ± 3.0	24.3 ± 3.2	<.001	22.8 ± 2.7	24.6 ± 2.6	<.001
WC	76.2 ± 8.8	83.7 ± 8.7	<.001			
Glucose	90.7 ± 7.8	97.8 ± 9.2	<.001	88.3 ± 9.9	96.5 ± 9.4	<.001
TC	184.0 [165.0; 202.0]	202.0 [183.0; 218.0]	<.001	187.0 [168.6; 206.2]	205.6 [189.4; 220.6]	<.001
TG	74.0 [56.0; 98.0]	162.0 [148.0; 179.0]	<.001	78.0 [59.0; 103.0]	161.0 [148.0; 177.0]	<.001
HDL	57.4 [52.0; 64.9]	50.6 [44.9; 56.4]	<.001	58.0 [51.0; 67.0]	50.0 [45.0; 56.0]	<.001
LDL	108.9 [92.6; 126.3]	117.6 [98.4; 132.1]	<.001	111.0 [93.0; 129.0]	122.0 [107.0; 136.0]	<.001

The high TyG index group comprises subjects with a TyG index ≥8.8

SBP, DBP, BMI, WC, Glucose are presented as the mean and standard deviation

TC, TG, HDL, and LDL are presented as the median, 1st quartile, and 3rd quartile

*KNHANES* Korean National Health and Nutrition Examination Survey, *KOICA* Korean Initiatives on Coronary Artery Calcification, *TyG* Triglyceride glucose, *SBP* Systolic blood pressure, *DBP* Diastolic blood pressure, *BMI* Body mass index, *WC* Waist circumference, *TC* Total cholesterol, *TG* Triglycerides, *HDL* High density lipoprotein, *LDL* Low density lipoprotein

WC as a data variable was only available in the KNHANES, and the high TyG index group had a significantly higher WC compared to the normal TyG index group (*P* < .001).

### TyG index according to smoking and alcohol consumption

**Fig. 2:**
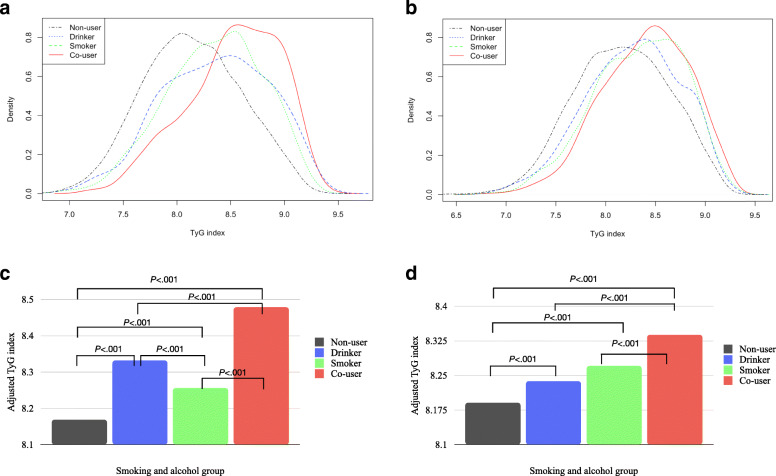
Difference of TyG index according to the smoking and alcohol groups. **a** Density plot of TyG index by group (KNHANES); **b** Density plot of TyG index by group (KOICA registry); Density plot means the distribution of a TyG index according to the S and A consumption groups. The co-user groups are widely distributed toward the higher TyG index. **c** Bar graph of TyG index after adjusting the covariates (KNHANES); d) Bar graph of TyG index after adjusting the covariates (KOICA registry); Values are presented as mean assessed by ANCOVA test; KNHANES model: adjusted for age, sex, final education, SBP, WC, TC, HDL, regular exercise, and S and A consumption; KOICA registry model: adjusted for age, sex, SBP, BMI, TC, HDL, regular exercise, and S and A consumption. KNHANES, Korean National Health and Nutrition Examination Survey; KOICA, Korean Initiatives on Coronary Artery Calcification; S and A, smoking and alcohol

The probability density distribution of the TyG index by the smoking and alcohol consumption groups is shown in Fig. 2a and b. In the KNHANES and KOICA registry, the distribution showed a greater shift toward the higher TyG index value in the “co-users” group than in the “non-users,” “drinkers,” and “smokers” groups.

After adjusting for the covariates, the TyG index value of the “co-users” group was significantly different from those of the other groups in both the KNHANES and KOICA registry (Fig. 2c and d).

### Risk factors for high TyG index

The results of the multivariate analyses and the predictors of high TyG index levels are presented in Table 3. Older age; male sex; higher SBP, WC, and TC; lower HDL; being in the “drinkers” group; being in the “smokers” group; and being in the “co-users” group were all significantly associated with a greater risk of a high TyG index in the KNHANES.

**Table 3:** Univariate and multivariate logistic regression analysis of risk variables associated with a high triglyceride glucose index

	KNHANES (2013–2018)				KOICA registry (2012–2016)			
	Univariate	*P*	Multivariate	*P*	Univariate	*P*	Multivariate	*P*
	OR (95 CI)		OR (95 CI)		OR (95 CI)		OR (95 CI)	
Age	1.02 (1.02–1.03)	<.001	1.01 (1.00–1.01)	.012	1.01 (1.00–1.01)	.102	1.00 (0.99–1.01)	.630
Sex								
Female	Reference [1]				Reference [1]			
Male	4.13 (3.61–4.73)	<.001	1.33 (1.10–1.59)	.002	6.20 (5.13–7.57)	<.001	2.13 (1.72–2.67)	<.001
Final education								
Elementary school	Reference [1]							
Middle school	0.77 (0.56–1.06)	.115	0.87 (0.89–1.56)	.425				
High school	0.70 (0.56–0.89)	.003	1.17 (0.61–1.23)	.266				
University	0.62 (0.50–0.78)	<.001	1.05 (0.79–1.40)	.743				
SBP	1.05 (1.04–1.06)	<.001	1.02 (1.02–1.03)	<.001	1.03 (1.02–1.03)	<.001	1.01 (1.00–1.02)	.002
WC	1.09 (1.08–1.10)	<.001	1.04 (1.03–1.04)	<.001				
BMI					1.25 (1.22–1.28)	<.001	1.09 (1.06–1.12)	<.001
TC	1.03 (1.02–1.03)	<.001	1.03 (1.03–1.03)	<.001	1.03 (1.03–1.03)	<.001	1.04 (1.03–1.04)	<.001
HDL	0.92 (0.91–0.93)	<.001	0.93 (0.92–0.94)	<.001	0.92 (0.91–0.93)	<.001	0.92 (0.92–0.93)	<.001
Regular exercise								
Yes	Reference [1]				Reference [1]			
No	1.09 (0.94–1.26)	.244	1.07 (0.91–1.26)	.412	1.18 (1.04–1.34)	.009	1.26 (1.09–1.45)	.002
Smoking and alcohol								
Non-user	Reference [1]							
Drinker	3.09 (2.30–4.10)	<.001	2.76 (1.97–3.84)	<.001	1.70 (1.41–2.03)	<.001	1.42 (1.16–1.73)	<.001
Smoker	2.37 (2.04–2.75)	<.001	1.56 (1.31–1.86)	<.001	1.80 (1.53–2.11)	<.001	1.33 (1.11–1.59)	.002
Co-user	4.59 (3.62–5.78)	<.001	4.33 (3.26–5.72)	<.001	1.94 (1.85–2.86)	<.001	1.94 (1.55–2.41)	<.001

The high TyG index group comprises subjects with a TyG index ≥8.8

*KNHANES* Korean National Health and Nutrition Examination Survey, *KOICA* Korean Initiatives on Coronary Artery Calcification, *OR* Odds ratio, *CI* Confidence interval, *SBP* Systolic blood pressure, *WC* Waist circumference, *BMI* Body mass index, *TC* Total cholesterol, *HDL* High density lipoprotein

Male sex; higher SBP, BMI, and TC; lower HDL; no regular exercise; being in the “drinkers” group; being in the “smokers” group; and being in the “co-users” group were all significantly associated with a greater risk of a high TyG index in the KOICA registry.

Compared to the OR (95% CI; *P* value) for the risk of high TyG index in the “non-users” group, the OR in the “drinkers” group was 2.76 (1.97–3.84; *P* < .001), in the “smokers” group was 1.56 (1.31–1.86; *P* < .001), and in the “co-users” group was 4.33 (3.26–5.72; *P* < .001) after adjusting for age, sex, highest education, SBP, WC, TC, HDL, and regular exercise in the KNHANES. Additionally, compared to the OR (95% CI; *P* value) for the risk of high TyG index in the “non-users” group, the OR in the “drinkers” group was 1.42 (1.16–1.73; *P* < .001), in the “smokers” group was 1.33 (1.11–1.59; *P* = .002), and in the “co-user” group was 1.94 (1.55–2.41; *P* < .001) after adjusting for age, sex, SBP, BMI, TC, HDL, and regular exercise in the KOICA registry.

### Evaluation of the predictive power of smoking and alcohol consumption

The results of the AUC comparisons, the NRI, and the IDI analyses are presented in Table 4. In the KNHANES, the AUC was larger when the smoking and alcohol consumption risk factors were considered together (without smoking and alcohol consumption, 0.817 [95% CI, 0.805–0.829]; with smoking and alcohol consumption, 0.829 [95% CI, 0.818–0.841]). The addition of smoking and alcohol consumption to the model without it significantly increased the AUC by 0.012 (*P* < .001). The NRI was estimated to be 0.040 and was statistically significant (*P* < .001). The IDI was estimated to be 0.017 and was statistically significant (*P* < .001).

**Table 4:** Evaluation of the predictive power of smoking and alcohol variables on a high TyG index

	KNHANES (2013–2018)		*P*	KOICA (2012–2016)		*P*
	Multivariate model	Multivariate model		Multivariate model	Multivariate model	
	Without S and A^a^	With S and A^b^		Without S and A^c^	With S and A^d^	
AUC (95 CI)	0.817 (0.805–0.829)	0.829 (0.818–0.841)	<.001	0.822 (0.811–0.834)	0.826 (0.815–0.838)	<.001
NRI (95 CI)	Reference	0.040 (0.014–0.050)	<.001	Reference	0.025 (0.007–0.043)	.006
IDI (95 CI)	Reference	0.017 (0.006–0.013)	<.001	Reference	0.005 (0.003–0.008)	<.001

^a^Without S and A model = age + sex + final education + SBP + WC + TC + HDL

^b^With S and A model = age + sex + final education + SBP + WC + TC + HDL + S and A

^c^Without S and A model = sex + SBP + BMI + TC + HDL + regular exercise

^d^Without S and A model = sex + SBP + BMI + TC + HDL + regular exercise + S and A

The high TyG index group comprises subjects with a TyG index ≥8.8

*KNHANES* Korean National Health and Nutrition Examination Survey, *KOICA* Korean Initiatives on Coronary Artery Calcification, *S and A* Smoking and alcohol consumption, *AUC* The area under the receiver operating characteristic (ROC) curve, *NRI* Net reclassification improvement, *IDI* Integrated discrimination improvement, *WC* Waist circumference, *BMI* Body mass index, *TC* Total cholesterol, *HDL* High density lipoprotein

In the KOICA registry, the AUC was larger when smoking and alcohol consumption risk factors were considered together (without smoking and alcohol consumption, 0.822 [95% CI, 0.811–0.834]; with smoking and alcohol consumption, 0.826 [95% CI, 0.815–0.838]). The addition of smoking and alcohol consumption to the model without smoking and alcohol consumption significantly increased the AUC by 0.004 (*P* < .001). The NRI was estimated to be 0.025 and was statistically significant (*P* = .006). The IDI was estimated to be 0.005 and was statistically significant (*P* < .001).

## Discussion

This study examined the effects of heavy smoking and alcohol consumption on the TyG index using two Korean population-based datasets. Smoking and alcohol consumption were independently associated with the TyG index after adjusting for confounding covariates. The TyG index mean values were significantly higher in the “co-users” group than in the “non-users,” “drinkers,” and “smokers” groups and were most likely to be ≥8.8. The AUC was also larger when smoking and alcohol consumption were considered together in both the KNHANES and KOICA registry. The estimated NRI and IDI values were statistically significant when smoking and alcohol consumption were considered together in both the KNHANES and KOICA registry.

Many studies have shown that TyG index predicts diabetes or cardiovascular disease. Sánchez-Íñigo et al. found that higher TyG levels were associated with and increased the risk of incident cardiovascular diseases in a Spanish population [4]. They reported that the HR in a group with a TyG index > 8.81 was 2.32 times higher than that in a group with a TyG ≤7.87 after adjusting for covariates [4]. In Korea, longitudinal studies also reported that elevated TyG index increases coronary artery calcification progression, a surrogate of cardiovascular disease [11, 36]. Lee et al. predicted the incidence of diabetes after 4 years through obesity and the TyG index in the Korean population [26]. Apart from the TyG index, LAP, TG:HDL cholesterol, and VAI have also been verified as surrogate markers for IR in previous studies [7, 37]. In KNHNES dataset, it was confirmed that the variables of LAP, VAI, and TG:HDL cholesterol were associated with smoking and alcohol consumption (data not shown). Finding and controlling risk factors for IR using simple IR surrogate indicators will help prevent future IR-related diseases.

Smoking is considered a major risk factor of metabolic diseases and CVDs [38]. Several studies also showed that smoking is associated with IR [17, 39]. Chronic smokers have a high incidence of IR and T2DM [40, 41]. Insulin responses to oral glucose load were significantly higher in smokers than in non-smokers [42], and insulin sensitivity was partially reversible after smoking cessation [43]. In a meta-analysis by Sun et al., which included 13 studies, current smokers had an increased risk of metabolic syndrome [44]. In line with previous studies, this study also found that smoking was independently associated with the TyG index, and the ORs for high TyG index were higher in smokers than in non-smokers. Tobacco extracts and smoke contain a large number of toxic materials that can produce various proinflammatory cytokines [40] that may augment oxidative stress, mitochondrial dysfunction, and inflammation, which may further contribute to decreased peripheral insulin sensitivity and insulin receptor affinity [17]. Smoking also causes direct impairment of β-cell function [39, 45].

The relationship between alcohol consumption, IR, and T2DM remains controversial and is dependent on the degree of alcohol consumption. Light to moderate alcohol consumption increases insulin sensitivity [21], and this increase is even higher with heavy alcohol consumption [20]. In a Korean cohort study, heavy alcohol consumption (≥30.0 g/day) was associated with a 1.3 times increase in the T2DM risk [46]. A meta-analysis evaluating the link between alcohol consumption and the risk of metabolic syndrome reported that heavy alcohol drinking (≥35.0 g/day) was associated with an increased risk of metabolic syndrome compared with non-drinking [47]. Consistent with findings from other studies, this study found that drinking was independently associated with the TyG indices, and the ORs for high TyG index were higher among heavy drinkers than among normal drinkers. There are a few possible explanations for this relationship. Alcohol abstainers, light drinkers (0.1–19.9 g/day), moderate drinkers (20.0–39.9 g/day), and heavy drinkers (≥40.0 g/day) had lower HOMA-IR levels than never-drinkers, and alcohol consumption was a major risk factor of β-cell dysfunction [48]. In addition, Jang et al. reported that chronic heavy alcohol consumption could potentially contribute to the development of T2DM by inducing β-cell dysfunction [49]. In rat models with chronic alcohol-related steatohepatitis, the histopathologic and ultrastructural abnormalities were associated with persistent hepatic IR, proinflammatory cytokine activation, and dysregulated lipid metabolism [19]. Therefore, chronic heavy drinking may adversely affect pancreatic β-cell function and activate inflammatory cytokines, which in turn may increase the risk of IR and metabolic disease. Further large-scale longitudinal studies are required to determine the effect of the degree of alcohol consumption on IR and T2DM.

Interestingly, this study found that smoking and alcohol consumption co-users had higher mean TyG indices than those who used alcohol or tobacco only. Furthermore, the predictive power of high TyG index was exacerbated when the two were combined. Previous epidemiological studies have demonstrated a high rate of concurrent use of alcohol and tobacco use [50], and health-related outcomes such as cancer, neurocognitive disorders, and increasing mortality were reported to be worse when smoking and alcohol consumption were combined [13–15, 51, 52]. Therefore, these results suggest that smoking cessation and control of heavy alcohol consumption could be effective in protecting individuals from IR-related chronic diseases. Moreover, if the results of this study are applied to co-users to emphasize the need for lifestyle correction, the effect of intervention or education will be greater. However, in this study, NRI, IDI, and the difference in AUC had statistical significance when smoking and alcohol consumption data were added but the values were low; therefore, the clinical interpretation of this findings could be considered weak. Further validation studies are required to better understand clinical implications and the pathophysiological of this findings.

In addition to smoking and alcohol consumption habits, male sex; higher SBP, WC, BMI; and lower HDL were risk factors of high TyG index. These results are consistent with those of previous studies on the associated factors of TyG index [26]. Factors associated with high TyG index should be considered in interventions to reduce insulin-related diseases. Notably, age and regular exercise showed inconsistent results between the two datasets. In previous studies, age [26] and regular exercise [36] did not show a unidirectional relationship with the TyG index. This analysis may have a selection bias, because the previous studies [26, 36] and the Korea registry targeted people with diseases or underwent general health examination, whereas the KNHANES was representative of Korean population. Further analysis of national data will be helpful to understand the relationship between TyG index, age, and regular exercise among the general population.

### Strengths and limitations

This study has several strengths. First, this study analyzed the data of a large, nationally representative sample of adult healthy Koreans. Second, the reliability of the results was improved by using two datasets. Furthermore, this is the first study to examine the combined effects of smoking and alcohol consumption on the TyG index.

Nevertheless, there were some limitations in this study. First, although data from two population-based studies were included, causal relationships between the exposures and study outcomes could not be established. Second, the information about smoking and alcohol consumption was self-reported, which may have resulted in reporting bias. Furthermore, a dose-dependent association between smoking and alcohol consumption and the TyG index could not be examined. Third, data regarding potential confounders such as dietary information could not be accessed. Fourth, the two datasets used in this study were not registered as clinical trials before the study began. Fifth, the design characteristics of the two datasets differed (e.g., cross-sectional study and retrospective study), and the definition of variables used in the analysis also differed. Sixth, since the missing data was less than 10% [53], the complete case analysis was performed, it was difficult to completely avoid selection bias. Finally, this study’s population was limited to Korean adults; hence, the results may not be generalizable to other ethnicities.

## Conclusion

In conclusion, smoking and alcohol consumption habits were independently associated with the TyG index, and co-users of alcohol and tobacco had higher TyG indices than who were non-users or those who exclusively consumed alcohol or smoked tobacco. Designing effective interventions and education programs on smoking cessation and adequate alcohol consumption should be considered to prevent increasing IR. In addition, concurrent users of smoking and high alcohol consumption are a target population that deserves more attention and concern in policy-based intervention for lifestyle modification.

## Data Availability

KNHANES data can be obtained from the Korea Centers for Disease Control and Prevention (http://www.cdc.go.kr/CDC/eng/main.jsp) after submission and evaluation of an appropriate research proposal. KOICA registry data analyzed during the current study are available from the corresponding author (H.J.C) on reasonable request.

## Abbreviations

AUC: Area under the area
BMI: Body mass index
BP: Blood pressure
CI: Confidence interval
CVD: Cardiovascular disease
DBP: Diastolic blood pressure
HDL: High density lipoprotein
HIEC: Hyperinsulinemic-euglycemic clamp
HOMA-IR: Homeostatic model assessment for insulin resistance
IDI: Integrated discrimination improvement
IR: Insulin resistance
KCDC: Korea Centers for Disease Control and Prevention
KNHANES: Korean National Health and Nutrition Examination Survey
KOICA: Korean Initiatives on Coronary Artery Calcification
LAP: Lipid accumulation product
LDL: Low density lipoprotein
NRI: Net reclassification improvement
OR: Odds ratio
QUICKI: Quantitative insulin sensitivity check index
ROC: Receiver operating characteristic
SBP: Systolic blood pressure
STROBE: Strengthening the reporting of observational studies in epidemiology
T2DM: Type 2 diabetes mellitus
TC: Total cholesterol
TG: Triglycerides
TyG: Triglycerides glucose
VAI: Visceral adiposity index
WC: Waist circumference
